# A Sustainable Extraction Approach of Phytochemicals from Date (*Phoenix dactylifera* L.) Fruit Cultivars Using Ultrasound-Assisted Deep Eutectic Solvent: A Comprehensive Study on Bioactivity and Phenolic Variability

**DOI:** 10.3390/antiox13020181

**Published:** 2024-01-31

**Authors:** Ouarda Djaoudene, Mostapha Bachir-Bey, Connie Schisano, Sabrina Djebari, Gian Carlo Tenore, Anabela Romano

**Affiliations:** 1 Centre de Recherche en Technologies Agroalimentaires, Route de Targa Ouzemmour, Campus Universitaire, Bejaia 06000, Algeria; 2 Laboratory of Applied Biochemistry, Department of Food Sciences, Faculty of Natural and Life Sciences, University of Bejaia, Bejaia 06000, Algeria; mostapha.bachirbey@univ-bejaia.dz; 3 Department of Pharmacy, University of Naples Federico II, 80131 Napoli, Italy; connie.schisano@unina.it (C.S.); gctenore@unina.it (G.C.T.); 4 Laboratory of Biomathematic, Biophysic, Biochemistry and Scientometry, Faculty of Natural and Life Sciences, University of Bejaia, Bejaia 06000, Algeria; sabrina.djebari@univ-bejaia.dz; 5 MED—Mediterranean Institute for Agriculture, Environment and Development, CHANGE—Global Change and Sustainability Institute, Faculdade de Ciências e Tecnologia, Universidade do Algarve, Campus de Gambelas, 8005-139 Faro, Portugal; aromano@ualg.pt

**Keywords:** natural deep eutectic solvents, phytochemicals, *Phoenix dactylifera* fruits, green extraction, antioxidant activity, enzyme inhibition, ultrasound-assisted extraction

## Abstract

The present study aimed to evaluate the efficacy of natural deep eutectic solvents (NADESs) on the extraction of phytochemicals from eight Algerian date fruit cultivars (*Phoenix dactylifera* L.). In this study, lactic acid/sucrose-based NADESs were used as an alternative to conventional chemical solvents using the ultrasound-assisted extraction (UAE) method. The obtained extracts were assessed for the determination of bioactive compound contents, phenolic composition, antioxidant activity, and enzyme inhibitory potential. The results showed a considerable variation in phytochemical compositions and related activities between cultivars, where the greatest contents of total phenolics (1288.7 mg GAE/100 g), total flavonoids (53.8 mg QE/100 g), proanthocyanidins (179.5 mg CE/g), and total triterpenoids (12.88 mg OAE/100 g) were detected in the fruits of the Ourous cultivar. The same cultivar displayed the highest antioxidant capacity against DPPH^•^ free radical (595 mg AAE/100 g), ABTS^•+^ cation radical (839 mg TE/100 g), and ferric reducing antioxidant potential (704 mg AAE/100 g). All extracts manifested moderate antioxidant activities tested by phosphomolybdenum, NO^•^, and linoleic acid lipid peroxidation assays. These extracts also exhibited interesting levels of in vitro enzyme inhibition; the Ourous cultivar gave the best inhibitory activity against α-amylase and acetylcholinesterase with 45 and 37%, respectively. HPLC-DAD-MS detected a total of five compounds, with phenolic acids and flavonoids being the main phenolics identified in the extract. The phenolic composition exhibited significant variability among cultivars. Notably, the highest amounts were revealed in the Tazizaout cultivar, with the predominance of gallic acid. The results confirmed that the combination of UAE and NADESs provides a novel and important alternative to chemical solvents for sustainable and environmentally friendly extraction and can represent a good alternative in food and pharmaceutical industry applications.

## 1. Introduction

Date palm (*Phoenix dactylifera* L.), a member of the *Arecaceae* family, represents one of the oldest cultivated trees in arid and semi-arid regions of the world, particularly in the Middle East and North Africa [1,2]. The production of dates is undoubtedly of great socio-economic and environmental importance. Algeria is the world’s fourth largest producer of dates, with around 1.18 million tons harvested in 2021 over a total area of 172 kha [3].

The fruit of the date palm is an important raw material, not only for its direct consumption but also for the many processing industries of transformation (powder, syrup, confectionery, juice, etc.). This fruit is highly consumed and appreciated for its high sugar content, source of fast energy, and antioxidant components such as phenolic compounds and vitamins [4].

In recent years, many researchers have studied the effects of date consumption on human health [5]. Moreover, several of these studies have also demonstrated the important effect of date polyphenols in the prevention of neurological diseases, cancer, diabetes, and cardiovascular, respiratory, and anti-inflammatory disorders [6,7,8,9].

Research on the bioactive compounds and nutraceutical properties of the date palm has highlighted the importance of the phenolic compounds and the possibility of using date pulp to develop functional food formulations with promising health potential [10,11].

The traditional organic solvents currently used in extraction processes are derived from non-renewable resources. These solvents are still widely used to extract natural compounds such as phytochemicals because of their powerful extraction and dissolving capabilities. Their usage is acknowledged to have adverse effects on the environment and to be detrimental to human health. Green solvents have emerged as an alternative to organic solvents due to their beneficial properties, such as reduced environmental impact and energy consumption during extractions, and the fact that the extracted materials can be safely used in various fields (pharmaceutical, cosmetic, and food industries) [12,13].

Several studies have investigated the extraction of phenolic compounds from date palm fruits [14,15]. However, despite the previously documented effects of conventional organic solvents, deep eutectic solvents (DESs) have never been used in the literature to extract phytochemicals from date fruits.

In recent research, there has been a significant focus on green and efficient deep eutectic solvents that can replace traditional harmful ones. DESs are considered a subclass of ionic liquids that are obtained from various renewable sources, such as biomass, thus offering many advantages, such as tunability, biodegradability, low toxicity, being more environmentally friendly, and simple preparation. Therefore, they can be used in many industries without any danger to human health [16,17]. These solvents are defined as a blend of two or more components, comprising a hydrogen bond acceptor and a hydrogen bond donor. Otherwise, DESs are able to donate and accept protons, allowing them to form hydrogen bonds with other compounds. The combination of components, when mixed in certain molar ratios, results in a mixture with a lower melting point and vapor pressure [18,19]. In this sense, mixtures of naturally occurring cations and anions prepared with components of natural origin, such as those obtained from natural organic acids, carbohydrates, amino acids, or natural compounds, like choline or betaine, are referred to as natural DESs [20,21]. The composition of NADESs can affect the efficiency of target compound extraction, as well as have a significant influence on physicochemical properties (solubility, conductivity, viscosity, density, and polarity). Consequently, they exhibit excellent solvation properties, potentially enhancing extraction efficiency and minimizing the required processing time [22,23]. Nevertheless, the choice of solvent and all of the above factors can be adjusted to improve the process’s extraction efficiency; therefore, the choice of the extracting technique is essential [21]. 

Ultrasound-assisted extraction relies primarily on ultrasonic energy to rupture cell walls, allowing solvents to penetrate the sample and release the target compounds. This method provides benefits compared to traditional extraction techniques, such as eco-friendliness, minimal energy, and solvent usage, and shorter extraction durations, which can be explored in more sustainable processes. In particular, ultrasound-assisted natural deep eutectic solvent extraction (UA-NADESE) has demonstrated good recovery rates, reproducibility, and high sensitivity [18,21].

Recently, studies have been published on extracting phenolic compounds from diverse biomass sources and botanical sources using the DES technique [17,24,25,26,27]. As far as we are aware, there are currently no published studies that examine the retrieval of phenolic compounds from date fruits using the UA-NADES method.

Therefore, the main objective of this work is to investigate sustainable alternatives to conventional chemical solvents for the extraction of phytochemicals. For this particular purpose, NADESs, which are environmentally friendly, combined with an innovative extraction technique, were evaluated for their efficacy in extracting bioactive compounds from different date fruit cultivars. To the best of our knowledge, this is the first study to investigate the analysis of the phytochemical composition and antioxidant properties of date fruits using green solvents and is the pioneering report to evaluate their enzyme inhibitory potentials against acetylcholinesterase (AChE) and α-amylase.

## 2. Materials and Methods

### 2.1. Chemicals and Reagents

Lactic acid (99% purity), Folin–Ciocalteu reagent, sucrose (98% purity), aluminum chloride (AlCl_3_ > 97% purity), and potassium persulfate (K_2_S_2_O_8_ > 99.99% purity) were purchased from Biochem-Chemopharma (Montreal, QC, Canada). Sodium carbonate (Na_2_CO_3_), sodium phosphate mono (NaH_2_PO), and dibasic (Na_2_HPO), as well as ascorbic acid (C_6_H_8_O_6_ > 99.53 purity) were obtained from Biochem-Chemopharma (Georgia, USA). Quercetin was procured from Panpharma (Shanghai, China). Ferric chloride (FeCl_3_) was from Panreac (Barcelona, Spain). The DPPH (2-2-diphenyl-1-picrylhydrazyl), ABTS (2,2-azinobis-3-ethylbenzothiazoline-6-sulfonic acid), and TPTZ (2,4,6-Tris (2-pyridyl)-1,3,5-triazine) were purchased from Sigma Chemical (Sigma-Aldrich, GmbH, Sternheim, Germany). Gallic acid was obtained from Prolabo (Montreuil, France). The DTNB (5,5′ dithiobis 2-nitrobenzoic acid) (>98% purity), acetylcholinesterase (Type VIS; EC 3.1.1.7), acetylthiocholine iodide, and porcine pancreatic α-amylase (EC 3.2.1.1, type VI) were from Sigma Chemical (Sigma-Aldrich, GmbH, St Louis, MO, USA).

### 2.2. Plant Material

Eight different cultivars of date palm fruits (known locally by Ourous “OUR”, Tazizaout “TAZ”, Tazarzeit “TAR”, Tazoughart “TAG”, Ouaouchet “OUC”, Oukasaba “OUK”, Delat “DEL”, and Tamezwertn’telet “TWT”) were harvested from M’zab oasis in the Ghardaia region (Southern Algeria). The selected mature fruits were of uniform size and free from physical damage, insects, injuries, and fungal infections. The moisture test indicated that these cultivars have a water content ranging between 11.34 and 39.04%. The fruits were pitted by hand, mixed using a blender, and stored in polyethylene boxes at 4 °C for further study.

### 2.3. NADES Preparation

The formulation of the used natural deep eutectic solvent was detailed in the study of Djaoudene et al. [28]. A mixture of lactic acid and sucrose, with a molar ratio of 3:1, was prepared in distilled water to aid dissolution. The mixture was then heated at 50 °C using a magnetic stirrer until a uniformly transparent liquid was achieved.

### 2.4. Extraction Procedure

Prior to implementing the experimental design, a preliminary investigation was conducted using a sequential methodology, as previously reported [28]. The objective of this step was to identify the factors influencing antioxidant extraction and establish the ranges of these influencing factors.

For the extraction procedure, crushed date fruits (0.2 g/15 mL) were mixed in the extraction solvent based on lactic acid and sucrose (3:1). The mixture was shaken for 15 min using a magnetic stirrer (VELP Scientifica, Usmate Velate, Italy) and then subjected to ultrasound-assisted extraction (power output of 130 W, frequency of 20 kHz, and probe dimensions of 138 mm × 3 mm) (Sonics Vibra Cell, VCX130PB, Newtown, CT, USA). Subsequently, the extracts underwent centrifugation at 5000× *g* for 15 min.

The solid-phase extraction column (Agilent, Bond Elut C18) was used to purify the phenolic compounds. The extracts were passed through the column and then reconstituted in methanol and formic acid (1%). The extract was then filtered using a nylon filter (Ø 0.45 µm) and stored at 4 °C for enzyme inhibition analysis and HPLC characterization.

**Figure 1:**
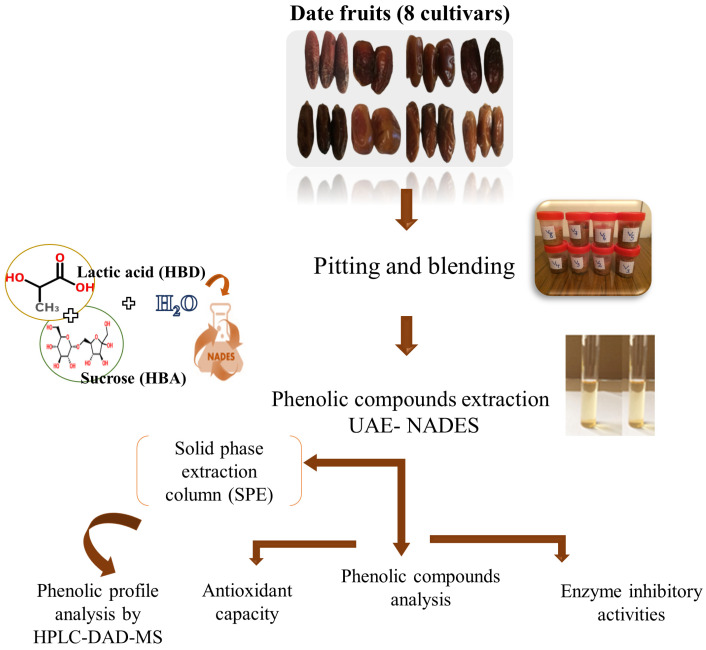
Scheme of the applied experimental approach followed in this study.

The various stages of the study are depicted in Figure 1 and detailed below.

### 2.5. Bioactive Compounds Analysis of Date Fruit NADES Extracts

#### 2.5.1. Determination of Total Phenolic (TPC) and Flavonoid Contents (TFC)

The determination of TPC was conducted through a spectrophotometric technique employing the Folin–Ciocalteu reagent, following the procedure outlined by Al-Farsi et al. [29]. A volume of 0.75 mL of diluted Folin–Ciocalteu reagent (10% *v*/*v*) was mixed with extract (0.1 mL) and sodium carbonate (0.75 mL, 6%). Following a one-hour incubation period at room temperature in the absence of light, the absorbance of the reaction mixture was recorded at 760 nm. The results were expressed as milligrams of gallic acid equivalents per 100 g of dry matter (mg GAE/100 g DM).

The total flavonoid content was determined according to the method of Quettier-Deleu et al. [30]. A volume of AlCl_3_ solution (0.75 mL, 2%) was mixed with an equal volume of extract. Following a 15 min incubation, the absorbance of the reaction mixture was assessed at 410 nm. The results were reported as milligrams of quercetin equivalents per 100 g of dry matter (mg QE/100 g DM).

#### 2.5.2. Determination of Proanthocyanidin (PA) and Total Triterpenoid Contents (TTC)

The proanthocyanidin content was estimated according to Škerget et al. [31]. The extract (200 μL) was homogenized with 3 mL of reagent consisting of FeSO4 at 0.54 mM prepared in a mixture of HCl-butanol (50:50, *v*/*v*). After incubation at 95 °C for 15 min, the absorbance was measured at 530 nm. The results were reported as milligrams of cyanidin equivalents per 100 g of dry matter (mg CE/100 g DM), utilizing the molar absorptivity coefficient of cyanidin (ε = 34,700 L/mol × cm).

The total triterpenoid content was assessed following the procedure outlined by Cao et al. [32]. Briefly, 500 µL of solution was added with 5 mL of reagent (5% *w*/*v* of vanillin prepared in glacial acetic acid) and 1 mL of HClO_4_ (perchloric acid). The mixture was subjected to incubation at 60 °C for 10 min, followed by cooling in an ice water bath for 15 min. Subsequently, 5 mL of glacial acetic acid was introduced and thoroughly mixed. The absorbance was measured at 538 nm after a 6 min duration. Oleanolic acid served as the reference standard, and the outcomes were presented as milligrams of oleanolic acid equivalents per 100 g of dry matter (mg OAE/100 g DM).

#### 2.5.3. Phenolic Profile of Date Fruit NADES Extracts

Liquid chromatography analysis was conducted utilizing a Jasco HPLC system from Tokyo (Japan) comprising a Jasco RHPLC pump (PU-4180), a Jasco photodiode array detector (MD-4010), a Jasco Intelligent Sampler (AS-2055 Plus), a Jasco column oven (CO-4061), a Jasco bottle stand (BS-4000-1), a Jasco LC-Net II/ADC interface box, and a Chrom NAV Control Center software (Version 1.18.04) connected to a computer system. The extracts underwent filtration using a membrane filter with a pore size of 0.45 µm. Subsequently, 20 μL of each sample was injected into a C18 Kinetex column (5 μm, 250 × 4.6 mm) held at a temperature of 25 °C. The analytical separation of phenolic compounds was performed according to the chromatographic procedure reported by Djaoudene et al. [33]. The eluent solvents, labeled as α and β, consisted of acidified distilled water (0.5% formic acid) and acetonitrile. The flow rate was set at 1.0 mL/min, and the elution gradient followed a linear pattern: starting at 10% β at 0 min, gradually increasing to 55% from 0 to 50 min, further elevating to 95% β from 50 to 65 min, reverting to 10% α from 65 to 70 min, and finalizing with column washing and re-equilibration. Identification of phenolic compounds involved comparison of their retention times and UV-vis spectra with pure phenolic standards. Quantification was carried out through external calibration curves utilizing peak areas of the obtained peaks at wavelengths of 280 and 360 nm. LOD (limits of detection) and LOQ (limits of quantification) were set based on taking 3 times the standard deviation (SD) above the mean blank value and 10 times the SD, respectively.

### 2.6. Antioxidant Capacity

#### 2.6.1. Ferric Reducing Antioxidant Potential (FRAP)

The FRAP evaluation was carried out according to Benzie and Strain [34]. For this, separate solutions of 10 mM TPTZ (dissolved in 0.04 M HCl), 0.005 M FeCl3, and sodium acetate buffer (0.3 M, pH 3.6) were prepared. The working reagent was then created by combining these three solutions at a volume ratio of 1:1:10. The reaction mixture consisted of 0.1 mL of extract and 0.9 mL of FRAP reagent. The absorbance was recorded at 593 nm after incubation at 37 °C for 30 min, and the results were reported as milligrams of ascorbic acid equivalents per 100 g of dry matter (mg AAE/100 g DM).

#### 2.6.2. DPPH^•^ and ABTS^•+^ Radicals Scavenging Assays

The free radical (DPPH^•^) scavenging assay was evaluated using the method reported in our previous study [28]. The extract (0.1 mL) was mixed with a fresh methanolic DPPH solution (1 mL, 60 µM). Following a 30 min incubation at room temperature in the absence of light, the absorbance was recorded at 517 nm. The DPPH scavenging activity was given as milligrams of ascorbic acid equivalents per 100 g of dry matter (mg AAE/100 g DM).

The ABTS radical cation decolorization assay was evaluated by referring to the method of Re et al. [35]. For the generation of the ABTS radical cation, a 7 mM ABTS stock solution was incubated with potassium persulfate for 14 h. The working solution of ABTS was obtained by dissolving the incubated solution in methanol until it reached an absorbance of 0.70 at 734 nm. The reagent solution (1 mL) was combined with the extract and subjected to incubation at 30 °C for 6 min, then absorbance was read at 734 nm. The ABTS scavenging activity was reported as milligrams of Trolox equivalents per 100 g of dry matter (mg TE/100 g DM).

#### 2.6.3. Nitric Oxide Radical Scavenging Assay

The ability of extracts to scavenge NO^•^ radicals was measured using the Greiss reaction as described by Sreejayan and Rao [36]. Briefly, the reactional mixture containing 500 µL of extract and 500 µL of 10 mM sodium nitroprusside (prepared in phosphate buffer at 20 mM, pH 7.4) was incubated for 2.5 h at 25 °C. Subsequently, 1 mL of Griess reagent was introduced into the reaction system, followed by a 30 min incubation, and the absorbance was recorded at 542 nm. The NO^•^ radical scavenging activity was reported as a percentage using the following equation: NO^•^ (%) = [(Ac − As)/Ac] × 100, where Ac and As are absorbances of the control (without extract but with the equivalent amount of extraction solvent) and the sample (with extract), respectively.

#### 2.6.4. Phosphomolybdenum Method (PhM)

The assessment of the total antioxidant capacity of the extracts was performed following the procedure outlined by Ramalakshmi et al. [37]. A 200 μL volume of the extract was introduced into 2 mL of the phosphomolybdenum solution (consisting of 600 mM sulfuric acid, 28 mM sodium phosphate, and 4 mM ammonium molybdate). The absorbance measurement of the mixture was recorded at 695 nm after incubation for 90 min at 95 °C. The results were reported as milligrams of gallic acid equivalents per 100 g of dry matter (mg GAE/100 g DM).

#### 2.6.5. Linoleic Acid Lipid Peroxidation Inhibition Activity (LALP)

Lipid peroxidation of linoleic acid was assessed as described by Gu et al. [38] with slight modifications. Briefly, the extract (200 µL) was mixed with linoleic acid (1 mL, 3% in ethanol), 2.5 mL of phosphate buffer (0.2 M, pH 7.4), and 1 mM FeCl_2_ (100 µL). Following incubation at 37 °C for 18 h, the reaction was halted by introducing 500 µL of 15% trichloroacetic acid (TCA) and 1 mL of 1% thiobarbituric acid (TBA). The reaction mixture was subjected to boiling for 15 min, subsequently cooled, and then extracted with 1 mL of n-butanol. The ability of the extract to inhibit lipid peroxidation was determined following this equation: inhibition (%) = [A_0_ − (A_1_ − A_2_)] ÷ A_0_ × 100; where A_1_, A_0_, and A_2_ are absorbances of the sample, the blank sample (without extract), and the blank control (without TCA and TBA), respectively.

### 2.7. Enzyme Inhibition Assay

#### 2.7.1. Acetylcholinesterase Inhibition Activity

The inhibition of acetylcholinesterase was conducted following the procedure of Ferreira et al. [39]. Succinctly, 275 μL of Tris buffer (pH 8; 50 mM), 100 μL of the extract, and 25 μL of enzyme solution (0.28 U/mL) were incubated for 10 min at 37 °C. After that, the reaction was initiated by adding acetylthiocholine iodide (AChI, 100 μL, 0.15 mM) and DTNB (500 μL, 3 mM). After a 5 min incubation, the absorbance was recorded at 405 nm. The results of AChE inhibition activity were reported as percentages, and galantamine was used as a reference inhibitor.

#### 2.7.2. α-Amylase Inhibition Activity

The α-amylase inhibition assay was carried out in accordance with the Sigma-Aldrich method, adapted from Ali et al. [40] with minor modifications. Briefly, 40 µL of the extract was mixed with distilled water (160 µL) and α-amylase solution (200 µL, 4 U/mL). After incubation for 10 min at 37 °C, 400 µL of starch (1%, *w*/*v*) was added. The reaction was started after 20 min of incubation at 37 °C and 200 µL of dinitrosalicylic acid color reagent solution was supplemented. The test tubes were incubated at 85 °C for 5 min, then the mixture was diluted by adding 2 mL of distilled water. The measurement of absorbance was performed at 540 nm, and acarbose was used as a standard reference. The α-amylase inhibitory activity was expressed as a percentage.

### 2.8. Statistical Analysis

All experiments were conducted in triplicate, and the presented data indicate the mean ± standard deviation for the entire set of results. Statistical analyses were performed using STATISTICA 8 software (Version 8.0.360.0) by analysis of variance (ANOVA-LSD test), and the relationship between different parameters and cultivars was evaluated using principal component analysis (PCA). A distinction was deemed statistically significant at a significance level of *p* ≤ 0.05.

## 3. Results and Discussion

### 3.1. Contents of Bioactive Compounds in NADES Date Extracts

The exploration of environmentally sustainable extraction processes has emerged as a prominent research focus in numerous applied chemistry domains. Therefore, deep eutectic solvents hold significant potential to be environmentally friendly and can substitute traditional solvents, given their comparable polarity to both water and organic solvents, which increases the range of potential applications for these solvents in the extraction of a variety of bioactive substances from food simples, herbs, and plants.

In this sense, the efficiency of US-NADESE investigated as a green alternative solvent for the extraction was evaluated in terms of its effect on total concentrations of polyphenols, flavonoids, proanthocyanidins, and triterpenoids extracted from the eight date fruit cultivars. As shown in Table 1, there was a significant variation in the antioxidant composition of the tested fruit cultivars, and OUR contained the significantly highest total phenolic and flavonoid contents (1288.7 mg GAE/100 g DM and 53.8 mg QE/100 g DM, respectively), compared to other cultivars. The proanthocyanidin and triterpenoid contents of the analyzed cultivars ranged, respectively, from 4.3 to 179.5 mg CE/100 g DM and from 5.28 to 12.88 mg OAE/100 g DM, and OUR contained the highest amounts.

**Figure 2:**
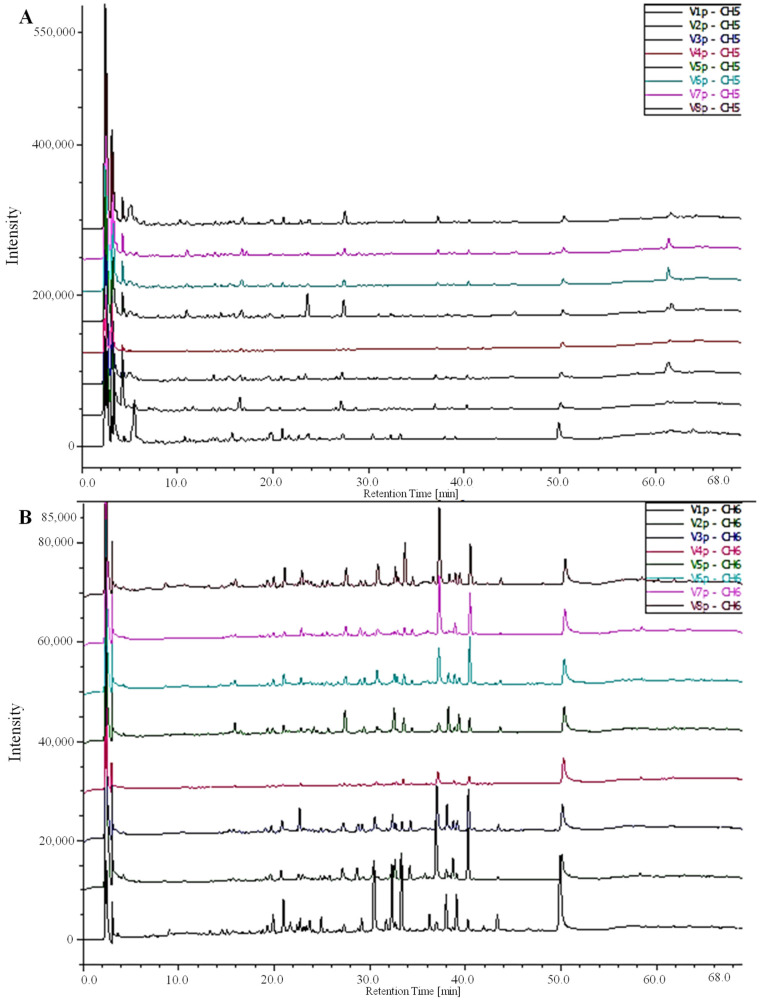
Chromatograms of HPLC-DAD-MS depicting the phenolic acids and flavonoid composition of green extracts from various fruit cultivars of *Phoenix dactylifera* L: (**A**) 280 nm and (**B**) 360 nm. V1: OUR; V2:TAZ; V3: TAR; V4: TAG; V5: OUC; V6: OUK; V7: DEL; V8: TWT.

The HPLC-DAD-MS results unveiled the individual phenolic compounds in date fruits in varying amounts, emphasizing the presence of five distinct phenolics, with three phenolic acids and two flavonoids (ferulic acid, vanillic acid, gallic acid, isoquercetin, and rutin) (Table 2). Ferulic and gallic acids were the main phenolics of OUC and TAZ extracts, while OUR extract showed a higher content of vanillic acid, isoquercetin, and rutin. The chromatographic profile of the detected phenolic acids and flavonoids of the different extracts is shown in Figure 2.

Comparison with the literature was challenging due to the novelty of this process. To the best of our knowledge, this is the first time that NADESs have been successfully used to extract phytochemicals from *Phoenix dactylifera* L. fruits. However, several authors have demonstrated the suitability of these solvents for the extraction of phytochemicals from various matrices. For example, Koutsoukos et al. [41] found that the use of DESs containing choline chloride: tartaric acid (2:1) resulted in about three to four times higher carotenoid yields from apricot pulp than using a mixture of methanol and chloroform. In another work, a series of NADESs (choline chloride/lactic acid, choline chloride/tartaric acid, lactic acid/glucose, etc.), were investigated as green alternatives to conventional solvents for the extraction of anthocyanins from fresh mulberry. The results showed a high yield of target compounds using chloride/citric acid/glucose (1:1:1) compared to conventional solvents [42].

The extraction of flavonoids from different types of fruits (fruits of *Lycium barbarum*, grape, plum, and cranberry) and vegetables (broccoli, onion) was assessed using various NADESs combined with ultrasound-assisted extraction [43]. The authors found that the best performance was obtained with acetylcholine chloride: lactic acid at 2:1, showing an efficient recovery of rutin, hesperidin, neohesperidin, naringenin, naringin, quercetin, hesperetin, and chrysin.

MAE (microwave-assisted extraction) combined with NADESs was used by Bajkacz and Adamek [44] as an extraction technique to isolate four isoflavones from flour, pasta, and breakfast cereals, namely daidzin, genistin, daidzein, and genistein. The authors found that the combination of ChCl: citric acid (1:1) gave the best yields. Another study reported by Kanberoglu et al. [45] evaluated the ability of DESs based on tetrabutylammonium chloride: decanoic acid (1:3) to extract quercetin from different fruits and vegetables, such as apples, grapes, onions, and tomatoes. Inspired by the previous study, NADESs based on lactic acid and glucose were evaluated for the phenolic compounds recovery from extra virgin olive oil [46]. Similarly, in the study conducted by Ivanovic et al. [47], the use of NADESs composed of choline chloride and lactic acid (1:2) was the most promising solvent for the extraction of phenolic compounds from *Lippia citriodora* leaves, achieving higher extraction yields than those obtained with 80% methanol.

The investigation performed by Macchioni et al. [48] on the ability of different lactic acid-based DESs to extract proanthocyanidins from hop (*Humulus lupulus* L.) using ultra-sonication revealed the effectiveness of the lactic acid: glycine system (3:1) for the recovery of these compounds, better than all tested DESs and conventional hydroalcoholic solvents. In this context, Neto et al. [49] underscored the effectiveness of combining deep eutectic solvents with microwave-assisted extraction for proanthocyanidin recovery from grape pomace, with the choline chloride and lactic acid mixture proving to be the most efficient.

The emerging green approach has also been used for the extraction of terpenoids. In a recent study, the response surface methodology was applied to maximize the extraction of triterpenoid saponins from *Xanthoceras sorbifolia* husks [32]. As a result, a mixture of tetrapropylammonium bromide and lactic acid (1:2) was selected as the best solvent, with extraction effectiveness up to 135% higher compared to 70% ethanol.

An earlier study similarly revealed an improved extraction yield by employing ChCl-LA at a molar ratio of 1:2 for phenolic recovery from the powder of date seed. Moreover, a total of eight phenolic compounds were detected, with 3,4-dihydroxybenzoic acid, catechin, and caffeic acid being the most predominant compounds [50].

Overall, this study demonstrated that the green solvent used is an interesting alternative solvent for the recovery of a broad spectrum of bioactive natural compounds from date fruits. It has been shown that the popularity of NADE solvents is related to their ease of penetration into the matrix and dissolution of the target compounds.

The effectiveness of NADESs for the extraction of biocompounds has been demonstrated by their properties and ability to exert a variety of interactions (hydrogen bonding, electrostatic) with bioactive analytes. Additionally, DESs are effective in disrupting the structures of biomass, leading to enhanced solubility and mass transfer of desired biomolecules [18,19].

### 3.2. Antioxidant Capacity of NADES Date Extracts

Numerous studies have indicated that bioactive compounds serve as the main contributors to the antioxidant potential in diverse foods and plant extracts, which often constitute highly complex multicomponent mixtures. These compounds exhibit diverse antioxidant mechanisms, including scavenging free radicals, reducing or chelating transition metal ions, and inhibiting lipid oxidation. It is therefore important to assess the antioxidant properties using several assays with different mechanisms of action.

In this study, it was measured using six different assays involving different mechanisms. The antioxidant activities of the date fruit extracts were assessed through FRAP, DPPH, ABTS, phosphomolybdenum (PhMo), NO^•^, and LALP. As reported in Table 3, the analyzed extracts showed strong antioxidant potential. The radical scavenging capacity of date fruit extracts, as determined by the DPPH and ABTS methods, ranged between 14.0–594.8 mg AAE/100 g DM and 125.4–838.7 mg TE/100 g DM, respectively. According to the results of the FRAP assay, OUR extract showed the highest reducing power with 704.2 mg AAE/100 g DM. In the PhMo assay, the antioxidant potential of different date fruit extracts ranged from 765.0 to 1229.0 mg GAE/100 g DM.

Antioxidant activity was also determined by monitoring the reduction in the NO radical. All the extracts tested showed moderate scavenging activity. Interestingly, the OUK extracts showed a significantly higher capacity (69.12%).

In addition, NADES extracts from date fruit were also able to inhibit lipid peroxidation, and the results showed that the OUK cultivar exhibited the highest inhibition activity (32.67%), with significant differences from most of the other cultivars.

All these results suggest that the NADES fruit extracts of the eight date cultivars showed significantly different results in terms of antioxidant activities; these variations may be due to the presence of different bioactive compounds as well as the synergistic effects of these components.

Due to the lack of studies on *Phoenix dactylifera*, it is not possible to directly compare these data with the literature, but there is nothing to prevent comparison with other studies that have used NADESs as extraction solvents. Therefore, a review of the relevant literature revealed that other authors have analyzed different matrices and biomass using NADESs or DESs as extractants. Kehili et al. [51] reported that the scavenging activities of date seed extract, obtained by microwave-assisted deep eutectic solvent using choline chloride/formic acid (1:3), were 27 (DPPH) and 145 (ABTS) mg TE/g dry biomass. Pan et al. [52] tested the antioxidant activity by DPPH, ABTS, and FRAP of Osmanthus fragrans flower extracts obtained with four DESs using microwave-assisted extraction and observed that the highest value was recorded for lactic acid: glucose (5:1). In another case, He et al. [53] evaluated the radical scavenging (DPPH) activity of NADES extracts from *Salvia miltiorrhiza*, and proline lactic acid extract showed significant effects against DPPH radical.

Similar outcomes were observed by Barbieri [54], who evaluated the antioxidant potential (DPPH and FRAP) of *Rosmarinus officinalis*; DES-based extracts presented the highest antioxidant capacity, with about three times more than ethanol extracts. A previous study also reported an improved efficiency of antioxidant potential (free radical scavenging activity and FRAP assay) when using ChCl/LA at a molar ratio of 1:2 for the recovery of phenolics from date seed powder [55]. The effect of NADES-based blueberry extracts using ChCl/glycerol/citric acid on ethanol-induced gastric ulcers has been reported previously, and the results showed a reduction in nitric oxide overproduction in treated rats [56].

### 3.3. Enzyme Inhibition Assay of NADES Date Extracts

The present study also investigated the biological properties of fruit extracts, including enzyme inhibitory activity. Indeed, Alzheimer’s disease (AD) is a progressive neurodegenerative disorder generally characterized by low levels of the neurotransmitter acetylcholine in the central nervous system. Currently, the only treatment options available to patients with AD are enzyme inhibitors, including acetylcholinesterase and butyrylcholinesterase. The mechanism of action of these cholinesterases is to interfere with the breakdown of acetylcholine, which alters cholinergic signaling [57].

Moreover, a key strategy for managing glycemia in patients diagnosed with type 2 and borderline diabetes is the suppression of the carbohydrate-digesting enzymes (α-glucosidase and α-amylase), as this dramatically reduces the postprandial rise in glycemia. Oxidative stress, which leads to the generation of reactive oxygen species, has also been linked to the onset and development of diabetes. Despite having undesirable side effects, conventional drugs such as acarbose, miglitol, and others used in practice are commercially available as enzyme inhibitors. Substances with antioxidant and anti-diabetic properties without serious side effects would therefore be very useful [58].

The enzyme inhibitory activity of the extracts was evaluated against AChE and α-amylase. The extracts demonstrated the ability to inhibit AChE and α-amylase with interesting levels of inhibition ranging from 18 to 37% and from 22 to 45%, respectively. Significant differences (*p* < 0.05) were found among the analyzed date cultivars; the results showed that OUR cultivar had the best inhibitory effect compared to other cultivars (Table 4). Galantamine and acarbose, commercially available enzyme inhibitors for AChE and α-amylase, were used as references, showing inhibitions of 76 and 79%, respectively.

Moreover, the extracts are very complex mixtures of compounds with distinct activities. The difference can be due to the synergistic or antagonistic effects of these compounds. The ability of the extracted bioactive compounds to inhibit physiological enzymes suggests that they have potential applications as neuroprotective and anti-hyperglycemic agents. Furthermore, this is the first time that the AChE and α-amylase inhibitory capacity of *Phoenix dactylifera* L. and NADES as extractants have been evaluated. Indeed, extracts obtained with different NADES from *Lavandula pedunculata* were found to be potent inhibitors of tyrosinase, acetylcholinesterase, and butyrylcholinesterase. The authors reported that the NADES extracts based on organic acids seem to contribute to significant AChE inhibition [48]. However, according to the authors, the acidifying agents (such as lactic, citric, and malic acids) can inactivate the enzyme activity by lowering the pH. The ability of DES-based *Ixora javanica* flower extracts to inhibit tyrosinase has also been described in the literature [59]. Another study reported that NADES-based *Cytinus hypocistis* extracts revealed interesting enzyme inhibition properties (cholinesterase, amylase, glucosidase, and tyrosinase) [60]. Fu et al. [61] found that *Carya cathayensis* Sarg. peels extracted with choline chloride–malic acid displayed significant inhibitory activity against α-glucosidase and α-amylase. Indeed, it has been previously proposed that NADESs play a role in modulating the activity of digestive enzymes [62].

### 3.4. Principal Compound Analysis

**Figure 3:**
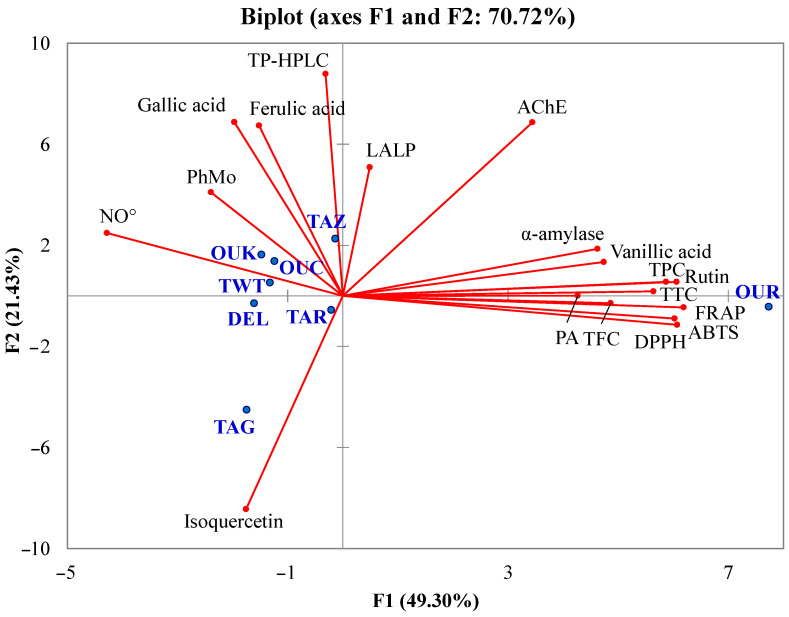
Biplot of principal component analysis. Analyzed parameters (axes in red), studied cultivars (blue points).

Principal component analysis is a statistical method used to transform and reduce multidimensional data into a smaller set of variables called principal components, capturing the maximum variance in the original dataset. In this study, the principal compound analysis was applied in order to check the presence of relationships between the different analyzed parameters, the individual and total phenolic content, and the antioxidant and enzyme inhibitory activities, to show the similarities between the studied varieties in a two-dimensional space. For the construction of this multivariate analysis, the measured parameters were taken into account, including antioxidant contents, phenolic compositions, antioxidant activities, and enzymatic inhibitions. The biplot representation gave a high cumulative variation of 70.72% that was divided between the two principal compounds, F1 (49.30%) and F2 (21.43%) (Figure 3).

The influence of total phenolics, individual phenolic compounds, antioxidant activity, and enzyme inhibitory properties was shown in the PCA biplot. TPC, TFC, PA, and TTC were highly correlated with FRAP, DPPH, and ABTS. Such a result is also in good agreement with Mansinhos et al. [48], who demonstrated the high positive influence of the total phenolic content and individual phenolic compounds on the antioxidant activity of *Lavandula pedunculata* subsp. lusitanica (Chaytor) Franco, evaluated by different assays (DPPH, FRAP, ABTS, and ORAC). The high correlations between the antioxidant activities assessed by various assays and the phytochemicals determined by spectrophotometric methods show that these substances are primarily responsible for the antioxidant capacity of date fruit extracts. Antioxidant activities evaluated by FRAP, ABTS, and DPPH assays were highly related to vanillic acid and rutin. Therefore, it is possible that these compounds may be one of the greatest contributors to the antioxidant potential of the *Phoenix dactylifera* L. extracts observed in this work.

The correlation analysis indicated that the main compound responsible for AChE inhibition was vanillic acid, which displayed a coefficient of correlation (r) of 0.75, while rutin was more linked to α-amylase inhibition (r = 0.73) and moderately to AChE inhibition (r = 0.57). Furthermore, the neuroprotective and anti-diabetic effects of flavonoids have been previously demonstrated [63,64]. Hence, rutin and vanillic acid are likely the primary contributors to the observed inhibition of enzyme activity by the extracts. In addition, it is possible that the contribution of some specific compounds or antagonistic and synergistic effects play an important role in the antioxidant and enzyme inhibitory capacities of the samples. Unlike to the present finding, in their previous study, Zengin et al. [60] observed that the total phenolic content of *Cytinus hypocistis* was highly related to the α-amylase inhibitory capacity. Therefore, the bioactivity of phenolic was more related to the composition rather than the amount due to the specificity of the mechanism of action as in the case of enzyme inhibition. In particular, the salt bridge or π-cation interaction involving the anionic carboxylate or aromatic ring and lysine residue of the enzyme (Lys200 for human α-amylase) is identified as crucial for the inhibitory activity of vanillic acid [65]. The study conducted by Oboh et al. [66] also indicated that flavonoids serve as effective inhibitors of α-amylase activity, with glycosylation further enhancing the inhibitory efficacy of quercetin. The authors demonstrated that the synergistic inhibitory effects on the enzyme were more pronounced when quercetin and rutin were combined than when each flavonoid was used individually. These findings align with the outcomes of the current study. Indeed, both TFC and rutin exhibited high correlations with α-amylase inhibitory activity, reaffirming the strong potential of flavonoids as promising candidates as α-amylase inhibitors. Considering all the performed analyses, the OUR variety was globally richer in antioxidants and more active, followed, respectively, by OUK, TAZ, TWT, TAR, OUC, DEL, and finally TAG.

## 4. Conclusions

In this study, the possibility of using NADES systems as a suitable alternative solvent based on lactic acid and sucrose was evaluated for the first time in the extraction of phytochemicals from date fruits. It was shown that this solvent, combined with UAE, is a very attractive approach to obtaining rich phenolic extracts with interesting biological activities. On the other hand, the cultivar factor was also shown to have a great influence on the content of both individual and total polyphenols, with the OUR cultivar showing the highest polyphenol content.

In addition, the sustainable extraction approach outlined in the study not only provides a technologically advanced method but also represents a promising and environmentally friendly method for obtaining valuable bioactive compounds from this natural source.

Overall, this innovative methodology holds the potential to contribute positively to both the scientific understanding of phytochemical extraction and the broader goal of sustainable and eco-friendly conscious research practices. Therefore, the exploration of alternative solvents and extraction techniques that facilitate the extraction of target compounds has a significant environmental and economic impact by proposing sustainable alternatives, reducing environmental pollution, and improving phytochemical recovery, especially when they are intended for use in the food, cosmetic, or pharmaceutical industries.

Further research based on in vivo and clinical studies is needed to confirm these extracts as viable food matrices with distinct biological properties. In parallel, it may also be interesting to investigate the stability of NADESs and gain insight into their physicochemical properties in order to understand their influence on extraction efficiency.

## Figures and Tables

**Table 1:** Antioxidant content of NADES extracts from date fruit.

Date Cultivar	TPC	TFC	PA	TTC
	(mg GAE/100 g)	(mg QE/100g )	(mg CE/100 g)	(mg OAE/100 g)
OUR	1288.7 ± 57.5 ^a^	53.8 ± 7.9 ^a^	179.5 ± 16.1 ^a^	12.88 ± 0.19 ^a^
TAZ	570.8 ± 20.7 ^cd^	40.3 ± 5.9 ^bc^	38.8 ± 2.2	9.72 ± 0.32 ^b^
TAR	502.3 ± 37.3 ^de^	44.8 ± 7.6 ^b^	51.1 ± 6.4 ^cd^	7.95 ± 0.36 ^c^
TAG	468.3 ± 59.7 ^e^	35.7 ± 3.4 ^cd^	38.0 ± 3.0 ^d^	6.52 ± 0.44 ^d^
OUC	444.0 ± 71.0 ^e^	28.0 ± 2.0 ^de^	4.3 ± 2.0 ^d^	5.52 ± 0.62 ^e^
OUK	663.8 ± 61.6 ^b^	39.3 ± 4.4 ^bc^	65.0 ± 8.2 ^c^	5.49 ± 0.82 ^e^
DEL	445.6± 53.2 ^e^	24.3 ± 1.0 ^e^	3.11 ± 0.12 ^d^	5.28 ± 0.15 ^e^
TWT	642.7± 33.1 ^bc^	34.5 + 6±4.0 ^cd^	153.3 ± 21.0 ^b^	5.99 ± 0.23 ^de^

Values labeled with distinct letters in the same column are considered significantly different (*p* < 0.05). TPC: total phenolic content; TFC: total flavonoid content; PA: proanthocyanidins; TTC: total triterpenoids content. GAE: gallic acid equivalent; QE: quercetin equivalent; CE: cyanidin equivalents OAE: oleanolic acid equivalent.

**Table 2:** HPLC-DAD-MS results for the individual phenolic composition of fruits of *Phoenix dactylifera* L. cultivars.

Date Cultivar	Phenolic Composition (mg/100 g DM)					
	Ferulic Acid	Vanillic Acid	Gallic Acid	Isoquercetin	Rutin	Total
OUR	3.534 ± 0.027 ^f^	4.378 ± 0.100 ^a^	2.160 ± 0.064 ^e^	0.020 ± 0.004 ^d^	5.503 ± 0.030 ^a^	15.817 ± 0.281 ^c^
TAZ	5.749 ± 0.306 ^c^	1.738 ± 0.173 ^c^	25.274 ± 3.401 ^a^	0.010 ± 0.002 ^e^	1.087 ± 0.032 ^c^	33.936 ± 3.932 ^a^
TAR	4.597 ± 0.055 ^d^	1.817 ± 0.074 ^c^	10.363 ± 0.436 ^cd^	0.020 ± 0.001 ^d^	0.884 ± 0.069 ^d^	17.692 ± 0.644 ^c^
TAG	1.093 ± 0.122 ^g^	0.541 ± 0.013 ^e^	2.091 ± 0.033 ^e^	0.240 ± 0.003 ^a^	0.569 ± 0.010 ^e^	4.296 ± 0.178 ^d^
OUC	10.175 ± 0.085 ^a^	1.113 ± 0.033 ^d^	8.609 ± 0.112 ^d^	nd	1.378 ± 0.049 ^b^	21.289 ± 0.289 ^b^
OUK	5.852 ± 0.252 ^c^	1.797 ± 0.076 ^c^	13.363 ± 0.886 ^b^	0.030 ± 0.005 ^c^	1.406 ± 0.054 ^b^	22.435 ± 1.268 ^b^
DEL	4.012 ± 0.063 ^e^	3.322 ± 0.070 ^b^	8.601 ± 0.470 ^d^	0.090 ± 0.011 ^b^	0.82 ± 0.018 ^d^	16.771 ± 0.631 ^c^
TWT	9.205 ± 0.127 ^b^	1.104 ± 0.042 ^d^	11.072 ± 0.328 ^c^	0.030 ± 0.002 ^c^	0.83 ± 0.048 ^d^	22.239 ± 0.555 ^b^
**Calibration Curves of Phenolic Compounds**						
Equation	Y = 5E + 07x − 10,514	Y = 6E + 06x − 1281.4	Y = 6E + 08x − 10,578	Y = 2E + 08x + 351.27	Y = 3E + 07x − 63.393	
LOD	0.0028	0.00885	0.001818	0.000858	0.002898	
LOQ	0.009334	0.0295	0.006061	0.002861	0.009659	
Linearity range (µg/mL)	0.25–12.5	1–50	1–50	0.25–12.5	0.25–12.5	

nd: not detected. Values labeled with distinct letters in the same column are considered significantly different (*p* < 0.05). LOD: limit of detection; LOQ: limit of quantification.

**Table 3:** Antioxidant potential of NADES extracts from *Phoenix dactylifera* L. date cultivars.

Date Cultivar	FRAP	DPPH^•^ Scavenging Activity	ABTS^•+^ Scavenging Activity	Phosphomolybdenum	NO^•^ Inhibition	LALP Inhibition
	(mg AAE/100 g DM)	(mg AAE/100 g DM)	(mg TE/100 g DM)	(mg GAE/100 g DM)	(%)	(%)
OUR	704.2 ± 19.2 ^a^	594.8 ± 37.4 ^a^	838.7 ± 34.0 ^a^	766.4 ± 33.7 ^e^	34.9 ± 2.7 ^e^	28.1 ± .1.0 ^abc^
TAZ	136.6 ± 8.2 ^b^	27.9 ± 8.6 ^e^	214.1 ± 19.3 ^b^	806.4 ± 52.2 ^e^	45.9 ± 1.6 ^d^	28.8 ± 2.0 ^cd^
TAR	137.4 ± 5.3 ^b^	59.6 ± 13.0 ^cd^	257.5 ± 13.3 ^bc^	765.0 ± 22.6 ^e^	49.3 ± 1.2 ^c^	24.3 ± 1.7 ^cd^
TAG	102.0 ± 7.9 ^c^	67.4 ± 10.7 ^c^	242.5 ± 17.5 ^bc^	775.2 ± 31.1 ^e^	48.7 ± 1.3 ^cd^	24.6 ± 3.3 ^cd^
OUC	107.5 ± 18.2 ^c^	46.0 ± 17.2 ^d^	183.9 ± 15.7 ^cd^	865.0 ± 32.6 ^d^	49.5 ± 2.7 ^c^	30.5 ± 1.7 ^ab^
OUK	119.2 ± 10.6 ^b^	52.7 ± 20.8 ^cd^	281.1 ± 30.5 ^bc^	1229.0 ± 45.6 ^a^	69.1 ± 1.4 ^a^	32.7 ± 6.1 ^a^
DEL	60.1 ± 23.1 ^d^	14.0 ± 7.5 ^e^	125.4 ± 20.5 ^d^	907.3 ± 26.2 ^c^	51.1 ± 2.2 ^c^	27.4 ± 5.1 ^bc^
TWT	109.2 ± 4.0 ^c^	80.0 ± 22.7 ^b^	249.2 ± 21.1 ^b^	1019.3 ± 40.2 ^b^	63.5 ± 4.9 ^b^	22.2 ± 3.3 ^d^

Values labeled with distinct letters in the same column are considered significantly different (*p* < 0.05). LALP: linoleic acid lipid peroxidation.

**Table 4:** Acetylcholinesterase and α-amylase inhibitory activities of date fruit NADES extracts.

Date Cultivar	AChE Inhibition (%)	α-Amylase Inhibition (%)
OUR	37.10 ± 0.97 ^a^	45.24 ± 0.36 ^a^
TAZ	34.28 ± 1.51 ^b^	33.25 ± 1.27 ^c^
TAR	29.44 ± 1.08 ^de^	30.37 ± 1.82 ^d^
TAG	18.41 ± 0.7 ^f^	24.81 ± 0.94 ^e^
OUC	31.48 ± 0.84 ^c^	40.61 ± 0.99 ^b^
OUK	30.38 ± 1.06 ^cd^	21.70 ± 1.06 ^f^
DEL	33.51 ± 1.89 ^b^	28.37 ± 1.21 ^d^
TWT	28.19 ± 0.59 ^e^	25.83 ± 2.57 ^e^
Galantamine (25 µg/mL)	76.69 ± 0.61	-
Acarbose (300 µg/mL)	-	79.60 ± 1.28

Values labeled with distinct letters in the same column are considered significantly different (*p* < 0.05).

## Data Availability

Data is contained within the article.
